# Cerebrospinal Fluid (CSF) CD8+ T-Cells That Express Interferon-Gamma Contribute to HIV Associated Neurocognitive Disorders (HAND)

**DOI:** 10.1371/journal.pone.0116526

**Published:** 2015-02-26

**Authors:** Rachel D. Schrier, Suzi Hong, Melanie Crescini, Ronald Ellis, Josué Pérez-Santiago, Celsa Spina, Scott Letendre, for the HNRP Group

**Affiliations:** 1 Department of Pathology, University of California San Diego, La Jolla, CA, United States of America; 2 Department of Psychiatry, University of California San Diego, La Jolla, CA, United States of America; 3 Department of Medicine, and University of California San Diego, La Jolla, CA, United States of America; 4 Department of Neuroscience, University of California San Diego, La Jolla, CA, United States of America; University of Cape Town, SOUTH AFRICA

## Abstract

**Background:**

HIV associated neurocognitive disorders (HAND) continue to affect cognition and everyday functioning despite anti-retroviral treatment (ART). Previous studies focused on mechanisms related to monocyte/macrophage mediated inflammation. However, in the ART era, there is increasing evidence for the involvement of CD8+ T-cells in CNS pathogenesis.

**Methods:**

To investigate the relationship between T-cell responses and neurocognitive impairment (NCI), cerebrospinal fluid (CSF) and peripheral blood CD4+ and CD8+ T-cell intracellular cytokine (IFNγ, IL-2, TNFα) and lytic marker (CD107a) expression were assessed in HIV infected subjects who underwent comprehensive neurocognitive (NC) evaluation and either initiated or changed ART.

**Results:**

Data were collected from 31 participants at 70 visits. The frequency of cytokine expressing T-cells in CSF was significantly higher than in peripheral blood for CD4+T-cells: TNFα, IL-2, IFNγ and CD8+T-cells: IL-2 and IFNγ. Analysis of T-cell activity and NCI as a function of CSF HIV RNA levels suggested a general association between NCI, high CSF CD8+ (but not CD4+T-cell) cytokine expression and CSF HIV RNA <10^3^ copies/ml (p<0.0001). Specifically, CSF CD8+ T-cell IFNγ expression correlated with severity of NCI (r = 0.57, p = 0.004). Multivariable analyses indicated that CSF CD8+T-cell IFNγ and myeloid activation (CD163) contributed equally and independently to cognitive status and a composite variable produced the strongest correlation with NCI (r = 0.83, p = 0.0001). In contrast, CD8+ cytolytic activity (CD107a expression) was negatively correlated with NCI (p = 0.05) but was dependent on CD4 levels >400/μl and low CSF HIV RNA levels (<10^3^ copies/ml). In our longitudinal analysis of 16 subjects, higher CSF CD8+IFNγ expression at baseline predicted NC decline at follow-up (p = 0.02). Severity of NCI at follow-up correlated with level of residual HIV RNA in CSF.

**Conclusions:**

Presence of IFNγ expressing CD8+ T-cells, absence of cytolytic CD8+ T-cells, high myeloid activation, and failure of ART to suppress HIV replication in CSF contribute to increased risk of HAND.

## Introduction

Although anti-retroviral treatment (ART) has dramatically reduced the incidence of HIV associated dementia, mild neurocognitive impairment (NCI) contributes to mortality and decreases quality of life of up to 40% of HIV infected individuals. Investigation of HIV associated NC disorders (HAND) has focused on myeloid cells (monocytes/macrophages/microglia) as the source of infectious HIV [1,2], HIV proteins and host inflammatory factors that mediate neuropathic damage via dendritic simplification, loss of synapses and ultimately neuronal loss[1,3,4,5,6,7]. However, correlations between clinical measures of NCI and markers of macrophage activation (neopterin, quinolinic acid, immunophillins, CD163, CD14) [8,9,10,11,12,13] are not robust in many study cohorts and fail to account for the association of NC impairment with low nadir CD4, higher levels of CXCL10 (chemotactic for T-cells) and presence of CD8+ T-cells expressing IFNγ in the CSF [14,15]. The consistency of these findings in diverse cohorts suggest that T-cells could play larger role in CNS pathogenesis and protection than is currently appreciated.

Assessing the role of T-cells in any HIV associated disease outcome is inherently complex due to the chronic nature of HIV infection and the central immune conflict of HIV disease: that HIV replicates in and depletes activated CD4 T-cells that are required to support anti viral CD8+ cytolytic (CTL) function and pathogen specific antibody production by B-cells. Thus CD4+ T-cell activation in the absence of ART is a double edged sword: it increases HIV virus production [16] [17], but also signals that the immune system is sufficiently intact to support pathogen specific antibody and cytolytic responses to a pathogen. CD8+ T-cell lytic activity appears to be unilaterally beneficial for the HIV infected host: sustained lytic function is associated with slower HIV disease progression, but in most HIV infected individuals, lytic function declines over time [18,19].

Genetic and epidemiological evidence suggests that the impact and correlates of T-cell responses to HIV in the CNS generally reflect those described for peripheral HIV viral control and pathogenesis, with some distinct differences: CD4+ T-cells are not present in the brain parenchyma and the brain is uniquely sensitive to inflammation [20,21]. Higher CD4+T-cell levels correlate with lower risk of HAND, possibly because exclusion of CD4+ T-cells from the CNS prevents them from contributing to HIV replication in the brain. Low (and HLA types that specify low) CD4+T-cell responses to HIV are associated with increased risk of HAND [17]. Low nadir CD4+ T-cells is also a risk factor for HAND [14,22], the proposed mechanism being that absence of CD4 cells reduces CD8 immune surveillance of CNS and lytic (CTL) function [23,24]. Genetic associations support a protective role of CD8+ T-cells: CD8+ HLA types that specify CTL responses to conserved HIV gag sequences [25,26,27] have a reduced risk of HAND [28], The suggested mechanism is that individuals with these genotypes maintain competent CTL (in the CNS) because viral escape mutations in conserved regions gag result in significant loss of viral fitness [24,29].

Despite the evidence above that higher levels of CD8+CTL and CD4+ T-cells are usually associated with reduce risk of HAND, T-cell mediated immune reconstitution syndromes (IRIS) have been described [3,30,31,32,33]. CD8+T-cell infiltrates appear to be the predominant pathogenic feature in brains from individuals who are on ART, not severely immunosuppressed, diagnosed with HIV infection of the CNS and undergo biopsy or come to autopsy [34,35]. However, it is not clear whether the CD8+T-cell infiltrates in IRIS cases are functionally or quantitatively different from the protective CTL responses described above. Also, the significance of the cellular infiltrate to cognitive outcomes is difficult to assess since few immunocompetent HIV infected brains undergo neuropathogenic analysis in the absence of pre-mortem symptoms. While our original studies in the pre-ART era failed to identify any T-cells in brain sections of patients with AIDS dementia [1], those findings do not preclude the presence of T-cells in the CNS at earlier, less immunocompromised stages of disease or in subjects on ART. It is of interest that post mortem staining of brains from asymptomatic SIV infected macaques indicates the presence of both CD8+ T-cell and NK cells with cytolytic potential (CD107a+) [21]. Since HIV infection of the Human CNS appears to occur early in disease in virtually all individuals, accompanied by high levels of MCP-1, CXCL10 [36,37] and CSF pleocytosis [38,39], T-cell infiltration may be more common than generally appreciated. In healthy individuals (not infected with HIV) a low level of lymphocyte trafficking is routine: CD8+ T-cells migrate into the brain to perform routine immune surveillance[40,41]. However, the high frequency of CSF pleocytosis and presence of lymphocyte infiltrates in tissue[42] suggest increased T-cell trafficking during HIV disease.

Although cell numbers in CSF are relatively low compared to peripheral blood, HIV associated pleocytosis provides an opportunity to characterize viable T-cells potentially in transit in or out of the CNS. While the utility of CSF biomarkers as a reflection of HIV pathogenesis in the brain was initially debated, accumulated data support the contention that CSF can be considered a “window” into the brain of the living host, especially during active HIV infection [36,43]. Studies of CSF lymphocyte subsets and activation states indicate that CSF T-cells from HIV infected individuals generally express high levels of surface activation (CD38+HLA-DR+), as well as chemokine receptors (CXCR3) and adhesion markers [37,44,45,46,47]. However, HIV specific CSF T-cell responses have not been previously investigated together with neurocognitive status. Using the UCSD associated HIV Neurobehavioral Research Program (HNRP) comprehensive neurocognitive battery for evaluation of HAND, including adjustment for norms, practice effects and metrics for change, we proposed to examine CSF T-cell responses in a cohort of subjects initiating or changing ART regimens. The goals of the project were to characterize cytokine expressing CD4+ and CD8+ T-cells in the CSF and their relationship to CSF HIV RNA levels and NCI. We hypothesized that: 1) Cytokine expressing T-cells in CSF might contribute to HAND, 2) Spontaneous T-cell cytokine expression would decline with suppression of CSF HIV RNA in most subjects, but 3) Persistent NCI would be associated with continued T-cell activation and might be enhanced in a subset of subjects with CD4 T-cell immune reconstitution.

## Methods

### Participants and Study Design

Eligibility criteria for the project included adults between 18 and 65 years of age, HIV seropositivity, intent to initiate or change ART, and willingness to undergo the study procedures (lumbar puncture, phlebotomy, neurocognitive testing). The project was approved by the University of California Human Subjects Protections Program. All 31 volunteers provided written informed consent. All subjects were medically and cognitively assessed prior to initiating or changing ART. The intent was to enroll 22 participants, 50% with NCI (GDS ≥0.5) and for all participants to be HIV RNA detectable (>40 copies/ml) at baseline. Study design included two additional visits (at week 6 and 16) following change or initiation of ART. Because of the modest budget for this exploratory (NIH R21) study, enrollment was linked to a parent treatment study. As a result of linked enrollment, 31 subjects were screened and underwent complete evaluation for this study but only 16/31 completed week 6 and 16 visits, for a total of 70 assessments in 31 adults. Visit number ranged from 1–3 visits per donor. Neurocognitive assessments were performed at enrollment and week 16.

At all visits, peripheral blood mononuclear cells (PBMCs) were isolated using CPT tubes (BD Vacutainer). CSF leukocytes were collected by centrifugation. PBMCs were drawn at all 70 visits and CSF T-cell intracellular cytokine (ICC) data were successfully acquired at 44 of the 70 visits, depending on whether lumbar puncture was successful and the number of cells recovered. Additional assessments at each visit included point-of-care urine drug screening, blood chemistry and complete blood count panels, HIV RNA quantification in CSF and plasma (Roche Amplicor version 1.5), and quantification of soluble biomarkers in plasma and CSF (TNFα, GM-CSF, IL-6, CCL2, CXCL10, soluble CD163, and soluble CD14 by either ELISA (R&D Systems) or bead suspension array (Millipore)).

### Intracellular cytokine assays

Measurement of intracellular cytokine expression after 6–24 hours culture with antigen is an established method of assessing T-cell responses to HIV and other viruses [48,49]. Cell culture, activation and antibody staining were done using a combination of protocols from BD FastImmune kits (BD Biosciences, San Jose, CA) and Lamoreaux et al[50]. All samples were fresh (not cryopreserved). After collection in CPT tubes (BD), PBMC were washed in phosphate buffered sailine (PBS) and resuspended in culture media (X VIVO 15 media with 5% Human Serum (Lonza)). After lumbar puncture, CSF cells were spun down, resuspended in culture media and counted in trypan blue to determine viability. PBMCs were plated at 100,000 cells per well and CSF cells at 5000–20,000 cells/well in V bottom 96 well plates in within 4 hours of collection. Cells were cultured with anti-CD28,49d co-stimulatory reagent (BD) and HIV p24 (Protein Sciences, 20 micrograms/well) or media as control. Brefelden A and Golgi Stop (both from BD) were added after 2 hours of culture and plates cultured overnight (18 hours) at 37C, 5% CO2. After culture, cells were washed (in plates) using staining buffer consisting of PBS supplemented with 0.1% bovine serum albumin (BSA) and 0.01% NaN3, fixed and permeabilized using BD Perm 2, and then stained using antibody cocktails: CD4-PerCp Cy5.5, CD8-APC, TNFα-PE, CD107a-FITC, IL-2-FITC, IFNγ-PE (all from BD biosciences) at 5–10% concentration depending on the antibody, in a total volume of 50μl. After 30 minutes incubation in the dark at room temperature, cells were washed with staining buffer described above, transferred to tubes, fixed with 1% paraformaldahyde and acquired on a FACS Calibur within 24 hours. Data were analyzed using a Flow-Jo (TreeStar) analysis template designed for the study. Gates for cytokine expression were synchronized in the template for each donor. Cytokine expression gates were defined based on PBMC unstimulated expression levels for each cytokine or CD107a and then applied to PBMC activated and all CSF files. Since constitutive cytokine expression was frequently higher in CSF than PBMC[51] and culture with antigen often reduced spontaneous cytokine expression, spontaneous cytokine expression was not subtracted from HIV p24 stimulated value, but remained in the database as “constitutive” cytokine expression. Frequency and absolute cell number data were both present in the database and used to evaluate significance of percentage. All cytokine expression data shown in tables and figures represent the square root of the percentage.

### Neuropsychologic Assessments

Participants underwent comprehensive neurocognitive, neuromedical evaluations and a structured psychiatric examination. Examinations were performed by trained and certified HNRP psychiatric staff at baseline and week 16 visits using standardized testing procedures. Details of the neurocognitive battery are provided in Cysique et al[52] and Heaton et al[53]. Neurocognitive impairment (NCI) status was defined by the global deficit score (GDS), a composite score that estimates functioning in multiple cognitive domains (Verbal Fluency, Attention/Working Memory, Speed of Information Processing, Learning, Delayed Recall, Executive Function, and Motor Function) and ranges from 0–5, with values above 0.5 representing clinically significant NCI. The GDS can be used as both a continuous and a binary variable[54]. Local norms for each GDS evaluation were used to account for practice effect and to classify significant change. Regression based change scores (for global NC impairment) were calculated for the 16 donors with longitudinal data, as previously described[55].

### Data Analysis Strategy

As described above, the intent of this study was to enroll a cohort (failing ART or ART naïve) to examine the relationship between CSF T-cell cytokine expression and NCI in the context of an anti-retroviral treatment study. However, the parent study screened and enrolled 8/31 individuals who were HIV RNA undetectable (<40 copies/ml) in both CSF and plasma at baseline[56]. Since T-cell responses and activation should be stratified according to exposure to antigen (in this case HIV) [30,37,57], the enrollment of HIV RNA suppressed individuals caused us to alter the overall analysis strategy according to presence of detectable HIV RNA, including week 6 and 16 as independent visits in the cross sectional analyses of immune responses. Inclusion of different numbers of visits per donor for was appropriate in this context because of the exploratory nature of the study, the scientific questions addressed, and the considerable effort invested to acquire the CSF T-cell cytokine response data. The degree of variation in number of visits was small (1–3/patient) and unlikely to lead to statistical bias. Studies of ex vivo T-cell responses/activation frequently include different numbers of evaluations when HIV RNA levels are dynamic, since within donor variation is usually greater than between donor variation and, in a dynamic system, additional data points contribute to a more accurate picture (See Antonelli et al and Shacklett et al). In this study, 3/16 longitudinal subjects had higher HIV RNA levels at week 6 or 16 than at baseline and we saw no rationale for excluding those visits from the HIV RNA detectable dataset. Cross sectional approach also allowed us to combine the HIV RNA undetectable visits at enrollment and follow-up. This policy was unlikely to lead to confounding over-representation from individual suppressed subjects since 2/4 of the baseline undetectable subjects became detectable at week 6 or 16 and those with sustained suppression usually had <20,000 T-cells in CSF. In contrast to immunologic assays, neurocognitive evaluations are performed In Vivo, subject to practice effect, do not vary directly with HIV RNA level, and are conventionally limited to a single evaluation per interval. NC assessments were only performed at enrollment and week 16 to minimize practice effects and biological data for week 6 were linked to week 16 NC scores. In respect to repeated measures concerns, and to demonstrate that the CD8+T-cell associations were significant using either a single or multiple visit analysis approach, the longitudinal analyses compared immunology and neuropsychology results using one visit at baseline and one week 16.

### Statistical Analyses

Statistical analyses were performed in JMP (version 9, SAS publishing, Cary, NC) and R. Variables with skewed distributions were transformed to improve symmetry. When transformation did not sufficiently improve distributions to meet assumptions for parametric statistical tests, non-parametric tests were used. For instance, between-group differences in intracellular cytokine expression or lytic marker levels was analyzed using a non-parametric two-tailed Mann-Whitney test or t-test depending on normality of the distribution. Comparisons with categorical variables with more than two levels were performed using analysis of variance. Paired tests were performed when comparing data from the blood and CSF compartments from the same individuals or when performing longitudinal analyses. Between-group comparisons of categorical data (e.g., detectable vs. undetectable HIV RNA) were performed using the Fisher’s Exact test. Linear regression was used to analyze the relationships between continuous variables.

### Ethics Statement

All samples and data were collected, assayed, and stored according to good clinical practice and to guidelines from the University of California, San Diego Human Subjects Protections Program. Written consent was obtained from all study participants. No adverse events were reported. Study ethics, including potential risks and benefits are an integral part of IRB approval at UCSD.

## Results

### Cohort: Demographic and Disease Characteristics

Cohort characteristics are shown in Table 1. Follow-up data were available for 16 of the 31 study participants after initiation (6) or change (10) in ART. Peripheral blood, HIV RNA levels and soluble cytokines were evaluated at 70 visits, with CSF T-cell cytokine data collected at 44/70 visits (the number of visits ranged from 1–3/participant). ART regimens were heterogenous, consisted of 3–6 antiretroviral agents and are described in the parent study [56]. As described above in the data analysis section, the original intent was to recruit HIV RNA detectable participants. However, the parent study enrolled nearly 1/3 HIV RNA undetectable (HIV RNA < 40 copies/ml) subjects. While enrollment of HIV RNA suppressed patients facilitated our ability to investigate mechanisms of immunopathogenesis when HIV RNA is suppressed, presence of both HIV RNA detectable and suppressed subjects at enrollment led us to stratify T-cell responses (contingent on presence of HIV proteins) across all visits except for longitudinal analyses.

**Table 1:** Cohort Characteristics.

Cohort	HIV+ Donors (n = 31)
Age	Median 48 (22–67*)
Edu Yrs	Median 14 (6–16)
Gender	97% M (1F)
CD4 plasma	Median 406/mm^3^ (58–1307)
Plasma HIV RNA	Median 50 c/ml (<40–148,242)
CSF HIV RNA	Median 44 c/ml (<40–64,606)
% HIV RNA<40	CSF&PL: 26% (8), CSF: 39% (12)
CSF cells	Median 4320/ml (110–27,400)
On ART	77% (24/31)
ART Naïve	23% (7/31)
GDS	Median 0.44 (0–1.72)
% GDS> = 0.5	45% (14/31)

*range

### Comparisons of T-cell cytokine expression in CSF and PBMC

**Fig 1:**
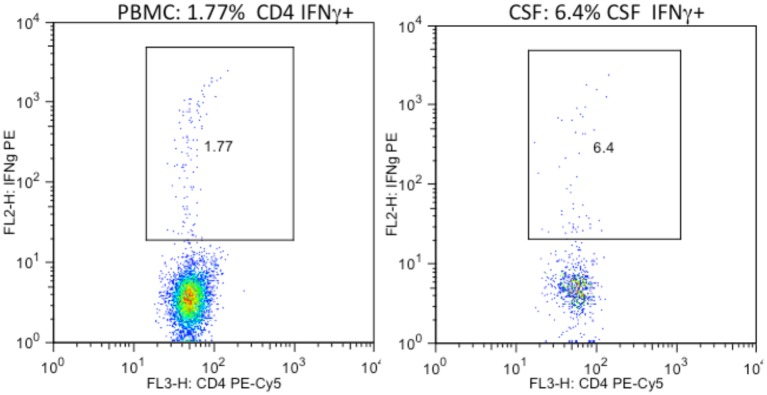
The frequency of HIV specific CD4 T-cells expressing IFNγ in the CSF was higher than in peripheral blood. PBMC (1a) and CSF (1b) cells from the same HIV+ donor were cultured overnight with HIV p24 antigen and stained for intracellular IFNγ expression.

A number of reports suggest that the frequency of activated and IFNγ expressing T-cells in the CSF is higher than in peripheral blood [37,45,51]. Comparisons of T-cell cytokine and lytic marker expression for our cohort are shown in Fig. 1 and Table 2. Fig. 1 shows paired HIV p24 induced CD4+ IFNγ responses in PBMC and CSF from a representative subject. Although the absolute number of cells in the CSF and the IFNγ gate is lower, the percentage of CD4 T-cells expressing IFNγ in CSF (6.4% in the gate) was more that triple the percentage in the blood (1.77%). The pseudo-color plots also illustrate that that the intensity of IFNγ expression was similar (Y axis) and the same gates could be used for CSF and peripheral blood. Comparison of CSF and PBMC constitutive and HIV p24 induced CD107a, TNFα, IL-2, and IFNγ expression at the 44 patient visits with CSF cell data is shown in Table 2. Median responses shown in the table were not transformed to illustrate actual frequencies, but significance testing used square-root transformed values and paired non-parametric two-tailed Mann-Whitney test. CD4+ T-cell IFNγ, TNFα, and IL-2 responses were significantly higher in CSF compared to blood. Constitutive CD4+CD107a expression was not higher in CSF, although the association trended in the same direction. CD8+ T-cell expression was higher in CSF for constitutive and HIV p24 induced IFNγ and IL-2, but not for lytic marker CD107a or constitutive TNFα.

**Table 2:** T-cell cytokine expression is often higher in CSF than in peripheral blood.

Condition	Cell/marker	Median% CSF	Median% PBMC	Wilcoxon (paired) (sqrt) * p =
Constitutive	CD4+CD107a+	0.53	0.32	0.2534
**HIVp24**	**CD4+CD107a+**	**0.69**	**0.30**	**0.0409**
**Constitutive**	**CD4+TNFα+**	**2.09**	**0.26**	**.0007**
**HIVp24**	**CD4+TNFα+**	**2.90**	**0.60**	**<.0001**
**Constitutive**	**CD4+IFNγ+**	**1.41**	**0.18**	**<.0001**
**HIVp24**	**CD4+IFNγ+**	**2.18**	**0.34**	**<.0001**
**Constitutive**	**CD4+IL-2+**	**2.30**	**0.39**	**<.0001**
**HIVp24**	**CD4+IL-2+**	**3.20**	**0.41**	**<.0001**
Constitutive	CD8+CD107a+	0.12	0.12	0.3575
HIVp24	CD8+CD107a+	0.32	0.23	0.4723
Constitutive	CD8+TNFα+	0.43	0.18	0.1616
**HIVp24**	**CD8+TNFα+**	**0.51**	**0.17**	**0.0474**
**Constitutive**	**CD8+IFNγ+**	**0.91**	**0.30**	**0.0007**
**HIVp24**	**CD8+IFNγ+**	**1.02**	**0.31**	**0.0002**
**Constitutive**	**CD8+Il-2+**	**1.48**	**0.27**	**0.0002**
**HIVp24**	**CD8+Il-2+**	**2.30**	**0.26**	**<.0001**

*For analysis, percent positive values were square-root transformed

### Association of NCI with CSF CD8+ T-cell cytokine expression at higher levels of CSF HIV RNA

**Fig 2:**
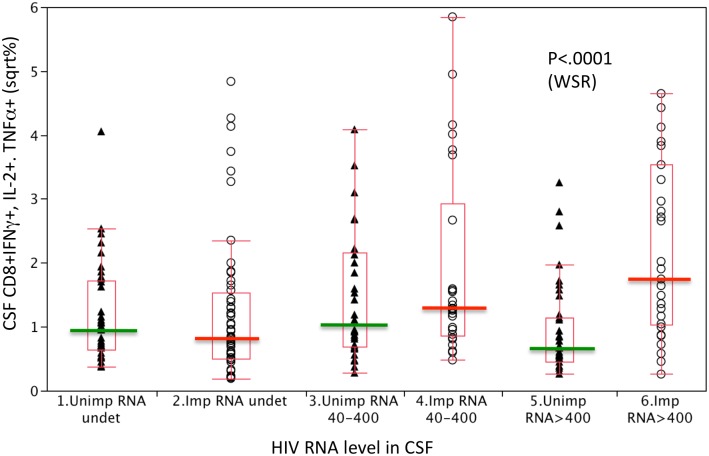
CD8+ T-cells from NC impaired subjects expressed increased levels of constitutive and p24 induced IFNγ, IL-2, and TNFα at higher CSF HIV RNA levels (>400 copies/ml). Constitutive and HIV p24 antigen induced CSF CD8+ IFNγ, IL-2, and TNFα expression by normal (triangles) and neurocognitive impaired (circles) subjects were compared at 3 CSF HIV RNA levels. Expression levels were square root transformed. Differences between impaired and unimpaired subjects were significant at higher HIV RNA levels (>400 copies/ml) in CSF (WRS = Wilcoxon Rank Sum p<0.001)

To investigate the relationship between CSF T-cell cytokine responses and HIV RNA level and determine whether NCI subjects differed from unimpaired subjects, CD4+ and CD8+ T-cell cytokine responses were compared at 3 different HIV RNA levels: undetectable, 40–400, and > 400. Selection of these categorical levels was based on the lower limit of quantitation (LLQ) for our HIV RNA assay (40 copies/ml) and the median HIV RNA level (381 copies/ml, or approximately 400) among subjects who had HIV RNA levels above the LLQ. Since preliminary analyses indicated that patterns were consistent for the 3 cytokines, CD8 T-cell TNFα, IFNγ, and IL-2 responses were combined (and not differentiated) in Fig. 2. Data groups were compared using a Wilcoxon Rank Sum test. Based on antigen dose response curves, we predicted a shallow bell shaped curve with peak responses at HIV RNA 40–400 copies/ml and lower responses when antigen (HIV) was undetectable or >400 c/ml. Such a pattern was observed for the non-impaired subjects (Fig. 2, triangles). However, the relationship differed for NC impaired subjects (circles): T-cell cytokine responses increased with HIV RNA level (<40 to >400c/ml, p = 0.0004). As a result, CD8 T-cell cytokine responses of participants with NCI were significantly higher compared to those of unimpaired participants at higher levels of CSF HIV RNA (>400 c/ml, p<0.0001). These findings suggest that NCI is more likely in individuals whose CD8+ T-cells respond to increasing HIV levels with correspondingly higher TNFα, IFNγ and IL-2 expression. This observation was specific to CD8+ T-cell cytokine responses: CD4+ T-cell cytokine responses of impaired and unimpaired subjects both increased as a function of CSF HIV RNA.

### Lymphocyte subsets, cytokine expression and soluble activation markers in blood and CSF cells from NCI and unimpaired participants

To identify characteristics of T-cell responses associated with NCI, all participant visits were separated on the basis of GDS score (≥0.5, <0.5) at date of visit and compared in Table 3. Neurocognitive impairment was significantly associated with lower CD4+ T-cell levels, higher levels of CD8+ T-cells, a low CD4+/CD8+ ratio, and higher/detectable HIV RNA levels in CSF. With respect to T-cell function, constitutive CD8+ TNFα, IFNγ, and IL-2 expression levels were each significantly higher at impaired visits, compared to non-impaired visits. In contrast, CD8+ CD107a (lytic marker) expression tended to be lower at impaired visits, although the association was not statistically significant. Neither soluble CD14 nor CD163 (markers of monocyte activation) were significantly higher in CSF or plasma at impaired visits.

**Table 3:** Neurocognitive impairment is associated with a low CD4/CD8 ratio in plasma and higher CD8+T-cell cytokine expression in CSF.

Measure (median)	Non-impaired	Impaired	t-Test
Visits	N = 42	N = 28	p =
GDS	0.183	0.657	------
**CD4 PL**	**471**	**406**	**0.027**
**CD4% PL**	**27**	**20**	**0.002**
**CD8 PL**	**778**	**1166**	**0.011**
**CD8% PL**	**44**	**59**	**<.001**
**CD4/CD8 PL**	**0.67**	**0.33**	**0.001**
On ART	32/42	20/28	.78 (FET)
**HIV RNA CSF**	**<40**	**48**	**0.035**
CSF RNA >40 (%)	22/42 (52%)	17/28 (61%)	.642 (FET)
HIV RNA PL	61	141	0.845
*sCD163 CSF pg/ml	5.4	6.3	0.264
* **cCD8+TNFα+ (%)**	**0.56**	**0.87**	**0.030**
* **cCD8+IFNγ+ (%)**	**0.86**	**1.06**	**0.010**
* **cCD8+Il-2+ (%)**	**0.95**	**1.83**	**0.053**
*cCD8+CD107a+**(%)**	0.64	0.22	0.155
*cCD8+CD107a+PB	0.60	0.39	0.076

*square root transformed

c = constitutive

All IC cytokine percentages are CSF unless marked “PB”

### Correlates of NC impairment when HIV is detectable in CSF

**Fig 3:**
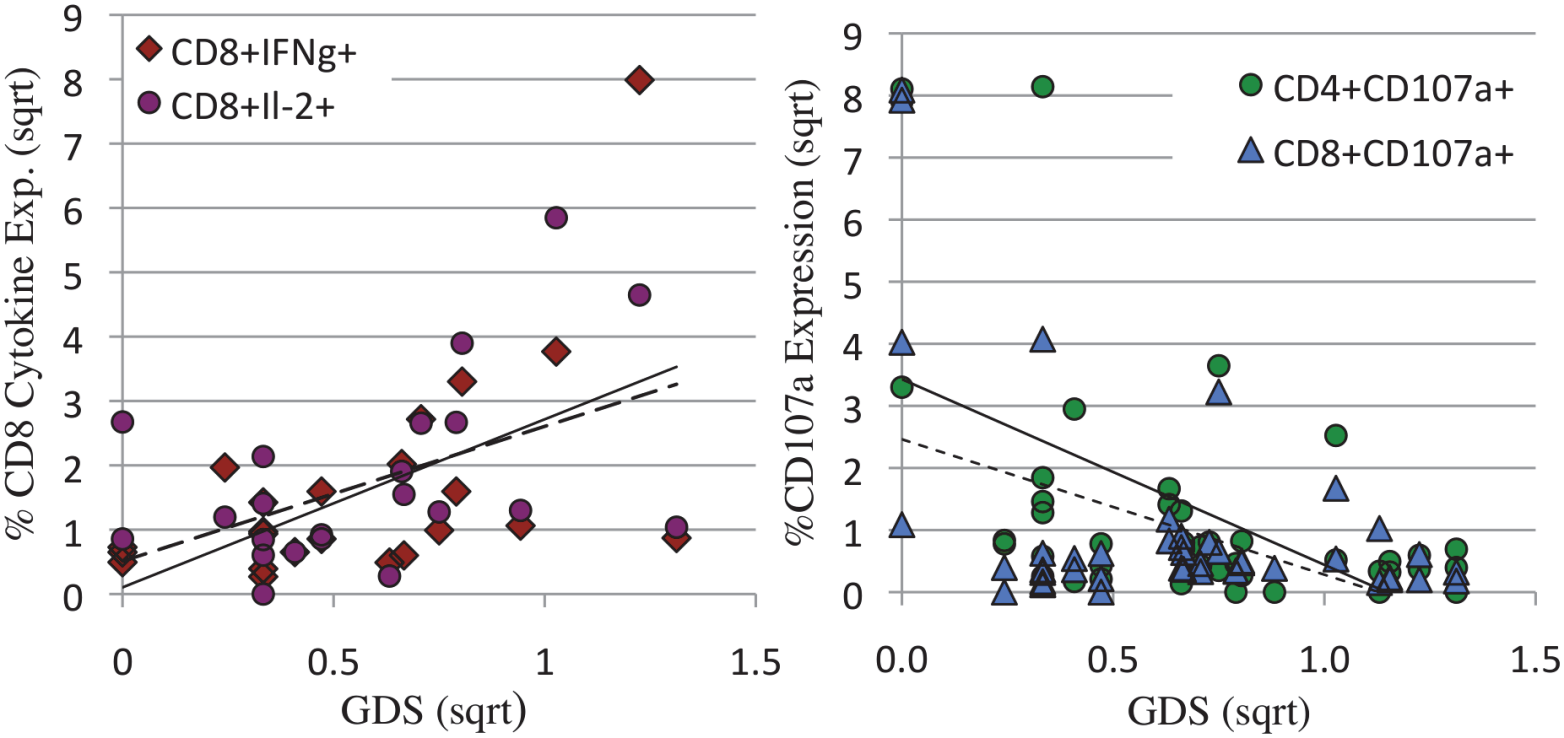
When HIV RNA was detectable in CSF, constitutive CD8+IFNγ and IL-2 expression correlated positively with NCI (Fig. 3a) and CD4+ and CD8+CD107a expression correlated negatively with NCI (3b) Percent cytokine (3a) or lytic marker (3b) expression were plotted as a function of neurocognitive global deficit score (GDS). Expression levels and GDS were square root transformed. **a**: When HIV RNA was detectable in CSF, there was a positive correlation between constitutive CSF CD8+IFNγ (solid line, diamonds r = 0.57, p = 0.004) and IL-2 expression (dotted line, circles r = .49, p = 0.01) and NCI. **b:** In contrast, CSF CD4+CD107a+ (solid line, triangles r = 0.49, p = 0.001) and CD8+CD107a (dotted line, circles r = .44, p = 0.004) expression were negatively associated with NCI.

**Fig 4:**
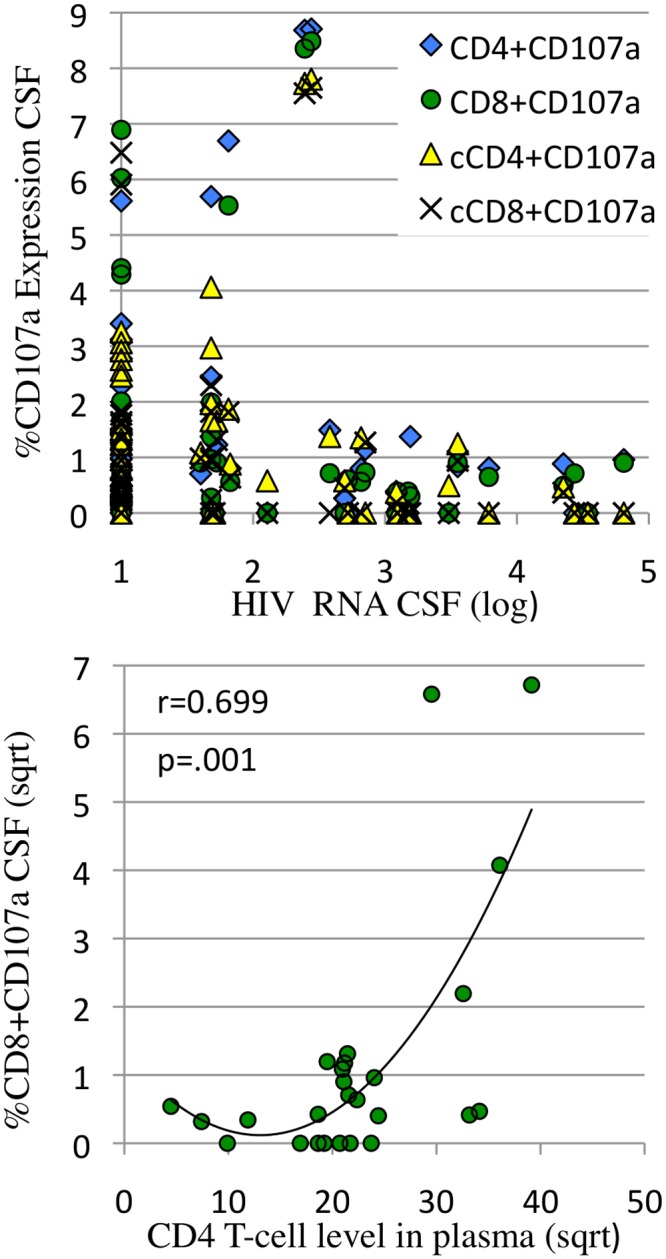
Constitutive and HIV p24 induced CD8+CD107a expression (lytic activity) in CSF and peripheral blood were only detected when HIV RNA levels were <1000 copies/ml and CD4 levels were >400/μl. **a:** The percentage of HIV p24 induced CD4+ (solid diamond) and CD8 (open circle) and constitutive (cCD107a) CD4+ (open triangle) and CD8+ (x) expression in CSF were plotted as a function of log HIV RNA in CSF. CD107a expression >1% was only detected when HIV RNA was <1000 copies/ml. **b:** The percentage of constitutive CD8+CD107a+ in CSF was plotted as a function of plasma CD4 levels. CD8+CD107a expression >1% was detected when CD4 levels were >400/μl. Cytokine expression and CD4 levels were square root transformed.

**Fig 5:**
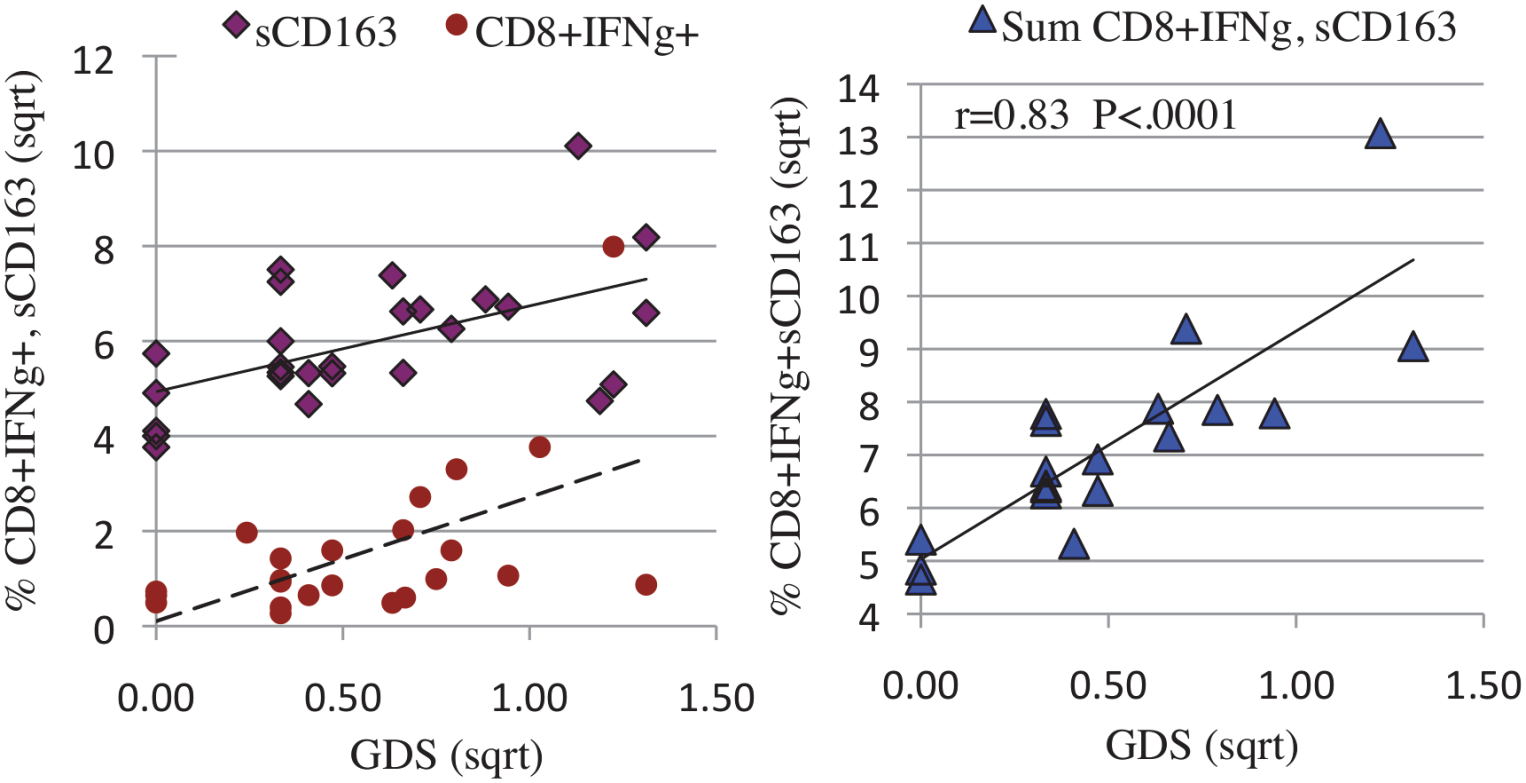
CD8+IFNγ + expression and soluble CD163 in CSF represent two significant and independent correlates of NCI (5a). The composite association is stronger than either alone (5b). **a:** The percentage of constitutive CSF CD8+IFNγ expression (solid circles, dotted line, r = 0.59 p = 0.004) and soluble CSF CD163 (open diamonds, solid line, r = 0.42, p = 0.004) were plotted as a function of neurocognitive global deficit score (GDS). **b:** The percentage of constitutive CSF CD8+IFNγ expression and pg of soluble CSF CD163 were summed for visits at which both values were available and the composite score plotted as a function of neurocognitive GDS (solid triangles, r = 0.83, p<0.0001). Expression levels and GDS were square root transformed.

**Fig 6:**
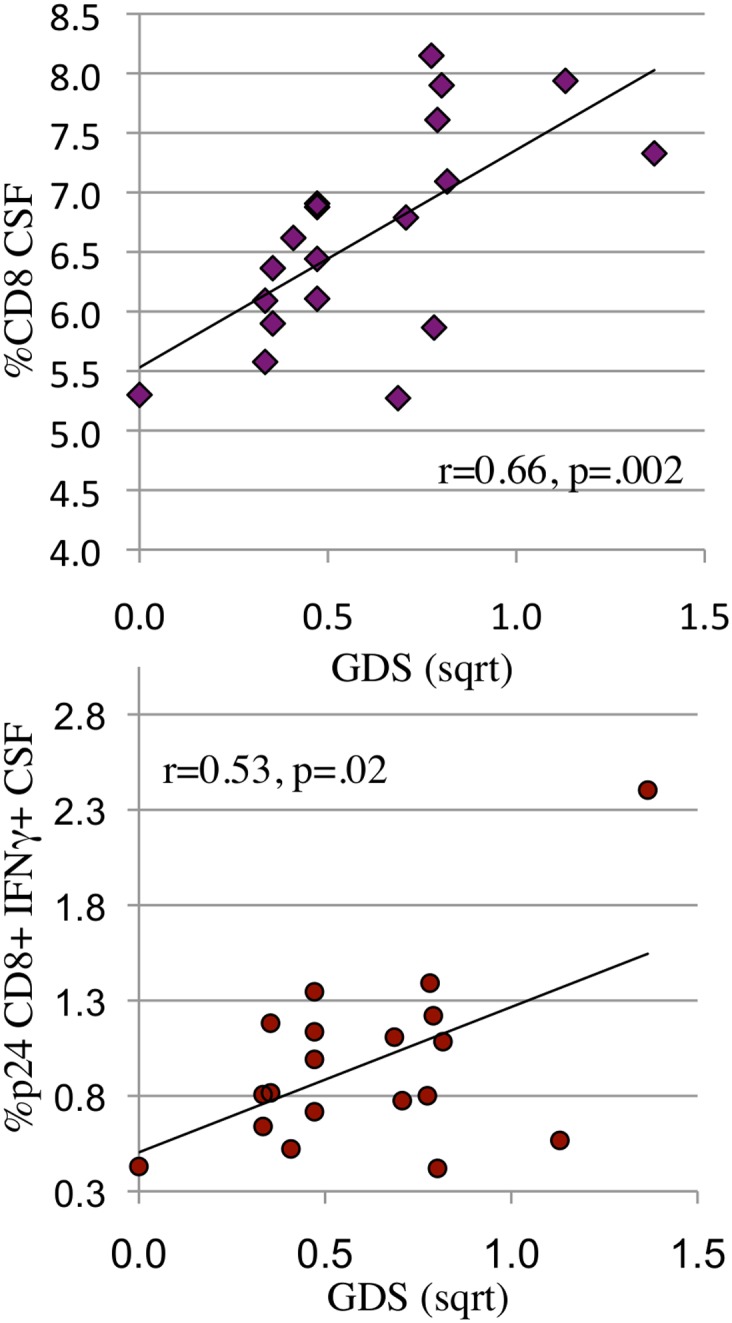
When HIV RNA is suppressed, the percent of CD8+ T-cells in CSF (6a) and the percent of HIV specific CD8+ IFNγ+ T-cells in CSF (6b) both correlate with severity of NCI (GDS). **a, b:** When HIV RNA was suppressed in CSF, CD8+ T-cells (6a: r = 0.66, p = 0.002) and HIV p24 induced CD8+ T-cell IFNg expression (6b: r = .53, p = 0.02) correlated positively with NCI (GDS). Cell numbers, expression levels and GDS were square root transformed.

T-cell cytokine expression within a 24 hour in vitro culture is dependent on recent exposure to HIV proteins and declines following suppressive ART. Dependence on HIV proteins predicts that associations between T-cell responses and NCI would be different in the HIV RNA/protein detectable and undetectable conditions. To address this prediction, CSF T-cell responses were examined at CSF HIV RNA detectable (> = 40 copies/ml) and undetectable (<40 copies/ml) visits, and T-cell cytokine and lytic markers correlated with NC function, using the GDS as a continuous variable within each data subset. Significant findings are shown in Table 4 and Figs. 3,4,5 and 6. Detectable HIV RNA in CSF was associated with: lower plasma CD4 T-cell levels, higher plasma CD8+ T-cell levels, lower plasma CD4/CD8 ratios, higher levels of CSF CXCL10, thought to be the major chemo-attractant for T-cells, and significantly higher levels of constitutive CD4+ and CD8+TNFα expression in the CSF (Table 4).

**Table 4:** Division of data according to CSF HIV RNA detectability reveals distinct CD8 T-cell and monocyte inflammatory responses correlations with NC impairment in the HIV RNA detectable and HIV RNA undetectable conditions.

Measure	HIV RNA>40	HIV RNA<40	t- Test
Total visit	N = 36	N = 34	p =
GDS median	0.44	0.44	0.479
**Age median**	**43**	**52**	**0.007**
**CD4 PL median**	**399**	**509**	**0.005**
**CD4% PL median**	**21.9**	**23.8**	**0.039**
**CD8% PL median**	**55.6**	**43.5**	**<0.001**
**CD4/CD8% PL median**	**0.4**	**0.6**	**0.007**
**On ART**	**20/36**	**31/34**	**0.001 (FET)**
**HIV RNA PL median**	**256**	**0**	**0.034**
**CXCL10 CSF median**	**2850**	**1411**	**0.006**
* **%CD4+TNFα+ median**	**2.3**	**1.5**	**0.050**
* **%CD8+TNFα+ median**	**1.0**	**0.7**	**0.034**
* **GDS vs sCD163**	**r = 0.42**	r = -0.01	**0.004**, 8**
* **GDS vs c.%CD8+IFNγ+**	**r = 0.59**	r = 0.33	**0.004**, 16**
* **GDS vs p24.%CD8+IFNγ+**	r = 0.05	**r = 0.53**	0.5, 0**.05** **

*values were square root transformed

** P value from ANOVA

Linear regression was used to identify the relationships between T-cell responses and GDS at CSF HIV RNA detectable and undetectable visits. While the level of NCI was not significantly different in the HIV detectable and undetectable conditions, the T-cell correlates differed. When HIV RNA was detectable in CSF, constitutive CSF CD8+ IFNγ, CD8+IL-2 expression and soluble CSF CD163 [58] correlated positively with worse NC functioning (Table 4, Fig. 3a). Since CD8+ IFNγ (or IL-2) and sCD163 did not correlate with one another: (R^2^ = 0.05) we hypothesized that the severity of neuroinflammation and cognitive dysfunction resulted from a combination of activated macrophages (sCD163) and CD8+ T-cell IFNγ expressing T-cells in the CNS (Fig. 5a), either a little of one and more of the other, or moderate levels of both. To evaluate this hypothesis, a composite variable was calculated by summing sCD163 and constitutive CD8+IFNγ expression. Plotting the composite variable against NCI (GDS) yielded a strong associated (r = 0.82, Fig. 5b). A further, multivariable analysis confirmed independence of sCD163 and constitutive CD8+IFNγ expression and their synergistic contribution to NCI p = 0.00004. While the correlation between severity of NC impairment and monocyte/macrophage activation was expected based on published evidence [8,58], the strong and independent contribution by CD8+ IFNγ + T-cells identified here suggests that CD8+ IFNγ+ T-cells cells play a central role in the neuropathogenesis of HAND.

In contrast to CD8+ IFNg expression, CD4+ and CD8+ T-cell CD107a lytic marker expression correlated with absence of NCI. (Table 4, Fig. 3b). We hypothesized that CD8+CD107a+ T-cells specifically lyse HIV infected cells in the brain, limiting HIV replication, without expression of inflammatory factors such as IFNγ. We also found that CD8+CD107+ responses were dependent on low HIV antigen levels (<3 logs in CSF, Fig. 4a) and higher CD4 T-cell levels in plasma >400/μl (Fig. 4b), both characteristic requirements of functional CD8+CTL [23,59].

### Correlates of NC function when HIV is undetectable in CSF

While HIV RNA suppression by ART halts and reverses CD4 loss, ART frequently fails to reverse HAND. One possible reason for persistent HAND is that some antiretroviral drugs don’t distribute into the CNS in therapeutic concentrations [60]. A non-mutually exclusive explanation is that, once initiated, neuopathogenic host responses become self perpetuating. To address the latter possibility, we asked whether specific CSF T-cell ratios or functions were associated with NC impairment when CSF HIV RNA was undetectable. The data shown in Table 4 (right column) and Table 5 represents cytokine expression data from 20 individuals who were HIV RNA undetectable in CSF at date of visit (24 visits). As shown in Table 4, the robust correlation between constitutive CSF CD8+ IFNγ expression and NC functioning weakened when CSF HIV RNA was undetectable. This would be expected: in the absence of HIV antigen, HIV specific CD8+ cells revert to a resting state and require re-exposure to HIV antigen to express cytokines. This expectation correctly predicted the significant correlation between HIV p24-induced CD8+ IFNγ expression and NC functioning at the CSF HIV RNA undetectable visits, shown in Table 4 and Fig. 6b. An association between percent of CD8+ T-cells in CSF and NC functioning was also detected (Fig. 6a). Together, the findings suggest that when HIV RNA is suppressed in CSF, NC impairment is related to a higher frequency of HIV specific CD8+ T-cells with the potential to migrate into the brain and express IFNγ should they encounter HIV proteins.

**Table 5:** When HIV RNA is suppressed, NC impairment is associated with higher CD8+ T-cell levels, higher CD14 levels, lower T-cell lytic activity and lower beta chemokine levels in CSF.

Measure	Non impaired (Median)	Impaired (Median)	t-Test p =
visits	N = 20	N = 10	
GDS	0.2	0.7	
CD4 PL	613	440	0.370
**CD8% PL**	**37.8**	**60.0**	**0.009**
On ART	18/20	10/10	0.54 (FET)
**sCD14 CSF(log)**	**4.94**	**5.19**	0.046**
*sCD163 CSF	5.4	6.1	0.764
* **CXCL10 CSF**	**1429**	**745**	**0.022**
* **CCL2 CSF**	**914**	**804**	**0.042**
* **CD8% CSF**	**6.1**	**7.6**	**0.006**
* **p24.CD4+CD107a+**	**1.3**	**0.4**	**0.051**
* **p24.CD8+CD107a+**	**0.9**	**0.3**	**0.026**

*Square root transformed,

**wilcoxon rank sum comparison

### Comparison of NCI and unimpaired visit T-cell responses when CSF HIV RNA is suppressed

To further characterize immune/inflammatory measures that persist despite effective ART, the HIV RNA undetectable visits were categorically divided into (NCI = GDS≥0.5) and unimpaired (GDS<0.5) visits (Table 5). Consistent with the linear association in Fig. 6a, NCI was categorically associated with higher CD8+ T-cell levels in CSF and plasma, suggesting that although HIV RNA is apparently suppressed, a relatively low CD4 T-cell level or ratio may perpetuate the pathogenic CD8+IFNγ phenotype. Higher CSF levels of CCL2 and CXCL10 were associated with the absence of NCI in these HIV RNA suppressed subjects, possibly related to the ability of these beta chemokines to attract HIV-p24 specific CD4+ and CD8+CD107a lytic cells (associated with less NCI in Table 5) into the brain. Myeloid cell activation markers sCD14 and sCD163 remained higher at impaired visits, although only sCD14 levels reached significance. In summary, the data suggest that when HIV RNA is suppressed, NCI is associated with lower levels of CXCL10 and CCL2.

### Relationship between CXCL10, NCI and types of T-cell responses

**Fig 7:**
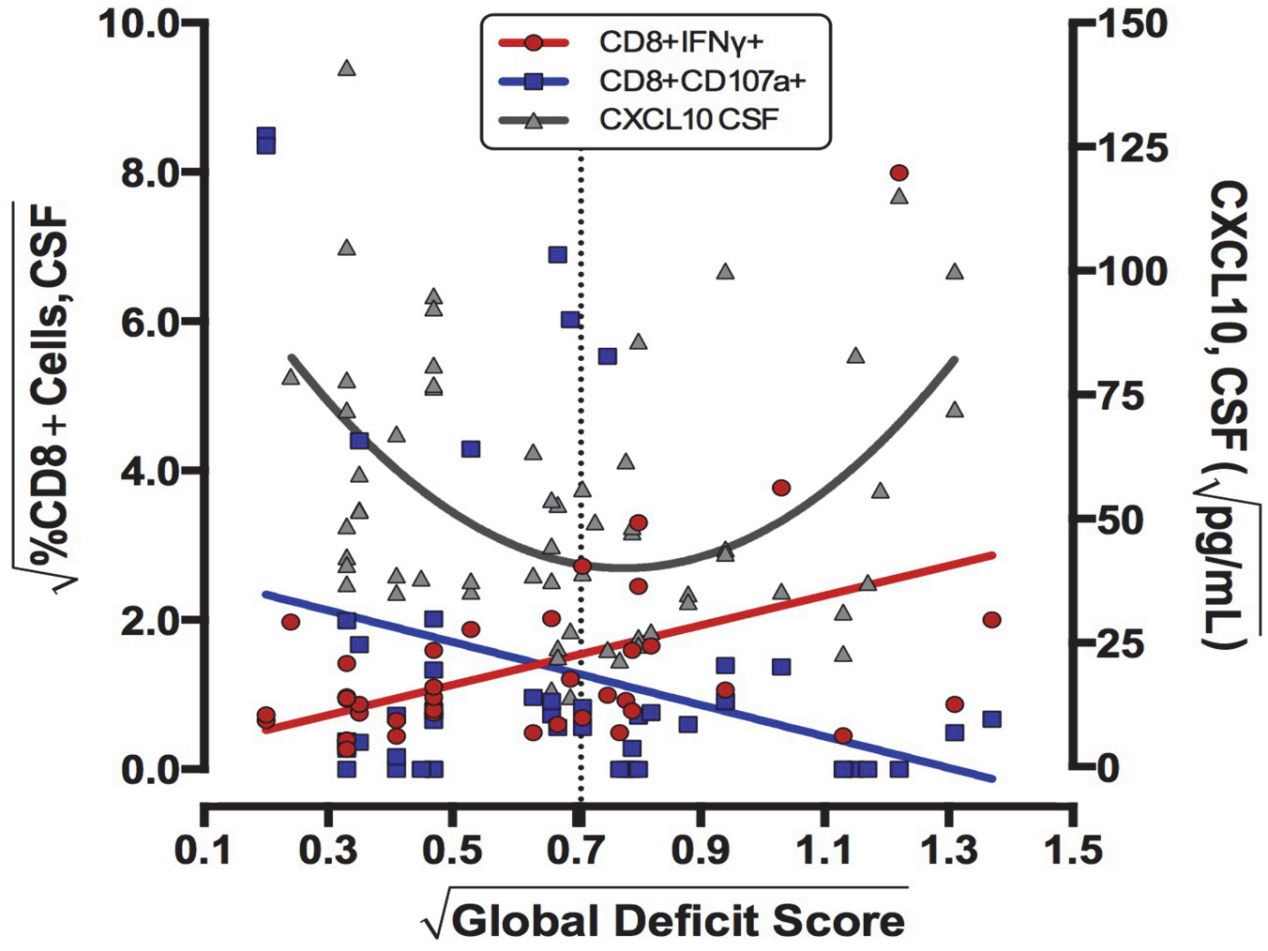
Neurocognitive outcome (GDS) can be viewed as a function of levels of chemoattractant (CXCL10) in CSF combined with the phenotype of CD8+ T-cells (CD107a or IFNγ+) available for recruitment into the CNS. Using all patient visits, best fit lines/curves for constitutive CSF CD8+CD107a (r = 0.28 p = 0.05), CD8+IFNγ expression (r = 0.47 p = 0.001) (both left axis), and CSF CXCL10 (r = 0.51 p = 0.004) (right axis) were plotted as a function of neurocognitive GDS. The dotted line indicates the conventional cut-off for clinically significant NCI, crossing near the inflection point for CXCL10 and the intersection of falling CD107a levels and rising CD8+IFNγ expression. CD8+T-cell expression levels, CXCL10, and GDS were square root transformed.

One goal of this study was to investigate the dynamics between beta chemokine levels and presence of T-cell populations into the CSF. Higher levels of CSF CXCL10 were associated with NCI when HIV RNA was detectable, consistent with CXCL10 mediated attraction of CD8+ IFNγ+ cells into the brain and NCI outcome. However, we also detected an association between absence of NCI, higher levels of CXCL10 and CD8+ CD107a expression when HIV was suppressed (Table 5). To better understand these seemingly contradictory findings, the data were viewed graphically by plotting best-fit curves for CXCL10, CD8+ IFNγ + and CD8+CD107a+ T-cells as a function of NC status (Fig. 7), with the dotted line indicating the cut-off for significant NCI. The best fit curve for CXCL10 shows high levels of CXCL10 are detected both in unimpaired and highly NCI states, the CXCL10 levels in unimpaired donors correlating with CD107a+ CD8+ T-cells and CXCL10 levels in impaired subjects correlating with CD8+ IFNγ expression. Viewed in this format, the data suggest that that both types of T-cells may be recruited into CSF by a CXCL10 gradient, with the effect on CNS inflammation and cognition depending on the functional phenotype (ie IFNγ or lytic CD107a) of CD8+T-cells available within the host.

### Effects of ART initiation/change on T-cell responses

We initially hypothesized that spontaneous T-cell cytokine expression would decline with suppression of CSF HIV RNA in most subjects, but persistent NCI would be associated with continued T-cell activation and might be enhanced in a subset of subjects with CD4 T-cell immune reconstitution. We attempted to address these questions by comparing baseline to week 16 evaluations, although the longitudinal analysis was limited to 16 subjects. The virologic status and T-cell levels are shown in the top half of Table 6. Effects of initiation (6) or change (10) of ART were predictable: HIV RNA levels declined and plasma CD4 levels rose at follow-up, but some changes did not reach significance, (probably a consequence of heterogeneous enrollment). GDS did not change between baseline and follow-up: six individuals met criteria for NCI at baseline and six at follow-up, with two becoming impaired and two becoming unimpaired. None of the subjects met criteria for immune reconstitution syndrome. Overall, baseline associations were similar to those detected in the HIV RNA detectable analysis (Table 4): there was a positive correlation between NCI and constitutive CSF CD8+ IFNγ + (r = 0.7, p = 0.01) and a negative correlation between CD107a lytic marker expression and severity of NCI (r = 0.45, p = 0.05).

**Table 6:** Longitudinal analysis confirms correlation between NC impairment and CD8+IFNγ+ (positive) and lytic activity (negative) at baseline and reveals positive association between NC impairment and residual HIV RNA in CSF at follow-up.

Median (N = 16)	Baseline	Follow-up:6,16	prt-Test p =
*GDS	0.33	0.44	0.487
HIV RNA CSF	509	0	0.109
**% HIV RNA CSF>40**	**69%**	**34%**	**.04 (FET)**
**HIV RNA PL**	**9488**	**48**	**0.0004**
% HIV RNA PL>40	69%	69%	NS
*CD163 CSF	5.5	5.5	0.972
CD4	385	465	0.299
CD8%	57	49	0.297
CXCL10 CSF	2891	1667	0.062
* **cCD8+IFNγ+%**	**0.56**	**0.41**	**0.003**
* **cCD8+Il-2+%**	**1.20**	**0.43**	**0.041**
*Regression (CSF)*	*r =*	*r =*	*Sig*.*ANOVA*
**GDS vs** * **cCD4+CD107a+**	**-0.45**	-0.08	**0.05**, NS
**GDS vs** * **cCD8+IFNγ+**	**0.70**	-0.05	**0.01**, NS
**GDS vs** * **p24.CD4+IFNγ+**	-0.05	**-0.39**	NS, **0.03**
**GDS vs** * **p24.CD4+TNFα+**	-0.15	**-0.42**	NS, **0.01**
**GDS vs** * **HIV RNA CSF**	.30	**.38**	NS, **0.02**

*c = constitutive*

*square-root transformed

Although plasma and CSF HIV RNA levels declined at follow-up, some subjects were incompletely suppressed and HIV RNA level in CSF correlated with severity of NCI at follow-up. As predicted, constitutive CD8+ IFNγ and IL-2 expression decreased at follow-up. Negative correlations between CD4+ TNFα, IFNγ, and NCI were observed at the follow-up visit, suggesting that increased CD4 activation following HIV RNA suppression might be associated with beneficial effects on cognition. Overall, the longitudinal findings confirmed the positive relationship between CD8+ IFNγ expression and the negative correlation between CD8+CD107a+ and NCI detected in the cross sectional analyses.

### Predictors of NC decline

Regression based change scores were calculated to identify predictors of NC decline for the 16 patients with longitudinal data. Eight of sixteen donors were neurocognitively stable, 2 improved and 6 declined. Baseline cytokine responses and clinical measures (HIV RNA, CD4, CD8+ levels) of “decliners” were compared to stable/improved study participants in Table 7. The only significant predictor of NC decline was higher HIV p24 induced CD8+ IFNγ expression. Decliners also trended toward having lower CD4+ T-cell percentages (p = 0.07).

**Table 7:** The most significant predictor of neurocognitive decline was higher CD8+IFNγ response to HIV p24 antigen at baseline.

Measure	Stable/ Improved (10) (BL Median)	Declined (6) (BL Median)	t-Test p =
CD4 in PL	450	317	0.12
CD4/CD8 in PL	0.44	0.27	0.18
*%CD4+ in CSF	5.4	3.6	0.07
CSF RNA >40	50%	100%	0.09
*cCD8+IFNγ+	0.69	1.79	0.10
*cCD8+Il-2+	0.85	2.66	0.10
* **p24.CD8+IFNγ+**	**0.73**	**1.56**	**0.02**

c = constitutive

*square root transformed

## Discussion

CSF and PBMC CD4+ and CD8+ T-cell spontaneous and antigen (HIV p24) induced intracellular cytokine and lytic marker expression were measured in a cohort of HIV infected study participants with defined NC status. T-cell responses were analyzed from several perspectives: First, CSF and PBMC cytokine responses were compared, showing that levels of CD4+ TNFα, IL-2, IFNγ, and CD107a and CD8+ IL-2, IFNγ, but not TNFα or CD107a were higher in CSF than in PBMC. This observation is consistent with published reports of elevated activation markers (CD38, HLA-DR, and CXCR3) on the surface of CSF cells from HIV infected individuals [37,44,47]. High levels of T-cell cytokine expression in CSF relative to PBMC may reflect increased migration of activated cells into compartments in general since genital tract T-cells are also more activated compared to those in the periphery[61]. Presence of CD8+ IFNγ expressing T-cells in CSF is emerging as a common mechanism of neuropathogenesis. Guinti et al[51] detected high spontaneous IFNγ expression by CSF CD8+T-cells from patients with a variety of neuroinflammatory diseases, including Multiple Sclerosis.

As expected, HIV antigen levels in CSF influenced levels of constitutive and HIV p24 induced T-cell cytokine responses. Peak CD8+T-cell cytokine responses of non-impaired subjects were detected at low HIV RNA levels and suppressed at CSF HIV RNA >400 copies/ml. In contrast, CSF CD8+ responses of NCI individuals increased with HIV RNA levels. The host or viral characteristics that enable T-cell cytokine expression in the face of high or persistent antigenemia remain to be identified, but could result from absence of regulatory T-cells, failure of other feedback mechanisms, or viral genotype. In contrast to cytokine response, lytic T-cell activity (CD8 CD107a expression) was suppressed at high HIV RNA levels in all subjects.

In cross sectional analysis, CSF T-cell cytokine responses were separated according to presence or absence of CSF HIV RNA (HIV antigen) at the visit date. CSF intracellular cytokine, lytic markers, and soluble biomarkers all tended to be higher when CSF HIV RNA was >40 copies/ml and significant correlations (r = 0.57) between constitutive CD8+ IFNγ expression and NCI were detected. The association between constitutive (rather than p24 induced) CD8+ T-cell response and NCI when HIV RNA was detectable is consistent with expectations: in the presence of active HIV replication, HIV specific CD8+ T-cells would be activated, expressing TNFα, IFNγ and IL-2 and would be detected as constitutive cytokine expressing CD8+ T-cells. T-cells already expressing cytokines are often refractory to additional HIV antigen induced activation, explaining the relatively weaker correlation between HIV p24 induced IFNγ expression and NCI when HIV RNA was detectable in CSF.

Myeloid cell activation in CSF, measured by sCD163, also correlated with NCI when HIV RNA was detectable (r = 0.54), consistent with reports from several groups[8,58]. Most significantly, addition of the myeloid CD163 and CD8+ IFNγ response data yielded a composite variable with a strong association with NCI (r = 0.82). This suggests that during ongoing HIV replication, both CD8+IFNg expressing T-cells and activated myeloid cells contribute to neuropathogenesis.

In contrast to CD8+IFNγ expression, CD107a expression was negatively correlated with severity of NCI. This neuroprotective association was detected for CD8+, CD4+, CSF and PBMC CD107a+ T-cell population responses. Since CD107a is a marker of cytolytic activity, our working hypothesis is that CD107a+ T-cells may be capable of killing HIV infected macrophages or microglia in the CNS with minimal inflammation and damage to bystander neurons and astrocytes (and no IFNγ expression). However, CD8+CD107a activity (>1%) was detectable under limited conditions: presence of higher CD4+ T-cell levels (>400 CD4/μl and low viral loads (<1000 copies/ml). Since CD8+ (and not CD4+) T-cells enter the brain, the requirement for CD4+ support for CD8+CD107a lytic activity may explain why low CD4+ T-cell nadir, high CD8 levels, and low CD4+/CD8+ ratio are often associated with NCI. Future studies will examine expression of other lytic markers: perforin, granzyme B and chemokine receptors on CSF T-cells [24,62,63].

Although this study is the first to differentiate between CD8+ T-cell phenotypes as either potentially protective (CD107a+) or pathogenic (IFNγ+) with respect to HAND, both observations are compatible with findings regarding systemic HIV disease status or progression. Cytoytic CD8+CD107a+, perforin, and granzyme B expressing cells are elevated in human “elite controllers” and destroy virus-infected cells in vitro [29,62,64]. Consistent with our findings that expression of CD8+CD107a was dependent on low virus load and a minimal CD4+T-cell level, evidence suggests that systemic T-cell cytolytic function is sensitive to both HIV RNA and CD4+ T-cell levels [59,65]. Our observations that low CD4/CD8 ration is associated with NCI is also consistent with systemic predictors of poor prognosis[66]. Although CD8+ IFNγ expression does not appear to be associated with systemic pathogenesis, there is a growing body of evidence indicating that it cannot be considered a correlate of viral control [29,62,67,68].

In spite of limited evidence concerning a role for T-cells in HAND, several studies identified IFNγ as an instigator of CNS pathogenesis. Soluble IFNγ has been detected in the CSF and CNS of HIV infected, NCI individuals[15,51] and can mediate toxicity in the CNS by inhibiting beta-catenin signaling in astrocytes [69] as well as by induction of CXCL10. In one study, HIV infection of mixed brain cell cultures In Vitro caused minimal neuronal toxicity, but addition of IFNγ triggered the CXCL10 pathway and neuronal death[15]. However, detection of IFNγ in CSF and brain of HIV infected humans has been controversial since myeloid cells and neurons do not make IFNγ and astrocytes only express IFNγ under ischemic stress [70]. Thus, critics have questioned studies implicating IFNγ because existing models of pathogenesis did not include a accepted cellular source. Presence of CD8+IFNγ+ T-cells in CSF, as described here, could explain the presence of IFNγ and we intend to stain for of CD8+IFNγ+ T-cells and NK cells in stored brain sections. In non-human primates, both CD8+T-cells and NK cells have been detected in brain tissue of SIV infected macaques [21].

Our findings presented here in no way contradict previous studies[1]. The initial focus on myeloid cells as the cellular source of neuropathogenic mediators was based on the absence of CD3+ T-cells in HIV Associated Dementia (HAD) brains autopsied in the pre ART era. Overt HAD occurred at advanced stages of immunosuppression when CD8+ as well as CD4+ T-cells were severely depleted. The absence of T-cells in immunodefficient HAD brains does not exclude potential involvement of T-cells in CNS pathogenesis of less immunosuppressed hosts. In fact, in the ART era, CD8+ T-cells have been the predominant infiltrating cell population detected in the CNS of patients who have come to autopsy diagnosed with HIV related immune reconstitution inflammatory syndrome (IRIS) [35,71,72,73,74]. The correlation between CD8+ T-cells and NCI identified in this study, considered together with detection of CD8+T-cells in the CNS in patients diagnosed with CNS IRIS raises the possibility that HIV associated neuropathogenesis diagnosed as IRIS or CD8+ T-cell encephalitis [75] might represent progressive migration of CD8+IFNγ+ T-cells into the CNS in a setting in which ART prevents severe immunosuppression, rather than a paradoxical response specifically triggered by ART. Future investigation will assess numbers and types of CD8+ T-cells in brain tissue from subjects with and without HAND to determine association with pre mortem NCI.

Lymphocytes are thought to be attracted into the CSF by viral antigens and beta chemokines secreted from as a consequence of HIV infection of the CNS. CXCL10, which attracts T-cells and monocytes expressing CXCR3 can be induced by IFNγ and is made by numerous cell types in the CNS, including microglia and astrocytes [76]. In our study, beta chemokines CXCL10 and CCL2 were present at higher levels in CSF when HIV RNA was detectable and there was a linear correlation between CXCL10 in CSF and CSF HIV RNA level (r = 0.44, p = 0.001). However the relationship between CSF CXCL10, T-cell responses, and NCI was complex. High CXCL10 levels were detected in CSF of individuals at both extremes of neurocognitive functional spectrum (minimally impaired and highly impaired), creating a U shaped curve relative to severity of NCI (GDS). Our interpretation is that if HIV is replicating in the CNS and eliciting high levels of CXCL10 in CSF relative to the periphery, then it is the qualitative function of available T-cells (CD107a+ lytic T-cells versus IFNγ inflammatory CD8+T-cells) that migrate along the CXCL10 gradient into the brain that determines whether the recruitment will be neuroprotective or neuropathogenic.

We investigated T-cell cytokine responses at patient visits when HIV RNA was undetectable (<40 copies/ml) to gain insight concerning persistent mechanisms of NCI when HIV replication was suppressed. While sustained NCI may indicate irreversibility of CNS damage, it may also reflect low level HIV replication or HIV protein expression in the brain. In CSF and plasma HIV RNA undetectable subjects, a low CD4/ CD8 ratio and presence of HIV specific CD8+ IFNγ T-cells in CSF was associated with worse NCI. The correlation of NCI with the HIV p24 induced (rather than constitutive) cytokine expression reflects consistency with virologic status of CSF: HIV p24 specific CD8+T-cells cells in CSF of an HIV suppressed patient would require exposure to HIV p24 antigen to express IFNγ. The observation that NCI was associated with HIV p24 induced CD8+ IFNγ levels in plasma HIV RNA undetectable hosts, reflecting how the HIV p24 specific CD8+T-cells would respond should they encounter HIV p24 in the brain, could be interpreted to suggest ongoing HIV protein expression in the CNS of those with sustained NCI.

A longitudinal analysis was performed for the 16 individuals with follow-up data after ART initiation or change. The baseline observations largely confirmed the cross-sectional analyses concerning positive and negative correlations between CD8+IFNγ or CD107a+ and NCI, respectively. A novel finding was that absence of NCI at follow-up was associated with increased p24 induced CD4+ T-cell cytokine (TNFα, IFNγ) expression in CSF. This negative relationship between CD4+ T-cell activity and NCI following ART is consistent with other studies indicating that functional CD4 reconstitution is a marker of good prognosis [14,17,22,28] [57]. In contrast to the absence of a direct relationship between HIV RNA and NCI in the cross sectional analyses, level of residual HIV RNA in CSF correlated significantly with severity of NCI at follow-up, confirming the importance of suppressing HIV RNA in the battle for control or prevention of HAND. However, the best baseline predictor of poor NC outcome at follow-up was higher HIV p24 induced CD8+IFNγ expression, suggesting that individuals whose CSF CD8+ T-cells make higher levels of IFNγ in response to HIV antigen may be at greatest risk of HAND.

A synthesis of the cross sectional and longitudinal analyses suggests the following potential scenarios during HIV infection: If HIV infects the CNS, eliciting CXCL10 when CD4 levels are high and virus levels low, HIV specific CD8+CD107a+ T-cells develop, migrate in response to CXCL10 and HIV antigens and traffic through the CNS, selectively destroying HIV infected myeloid cells and controlling HIV replication with minimal cerebral inflammation. However, with persistent viral replication, CD4+ T-cell levels decline and no longer provide systemic support for CD8+ lytic activity. Without CD4+ T-cell support, CD8+ T-cell function is limited to cytokine expression, changing the phenotype of the recruited T-cells from lytic to IFNγ expressing CD8+T-cells. In the confines of the CNS, IFNγ expression is toxic (especially in combination with HIV proteins) eliciting additional CXCL10 and CCL2[8,15,36], attracting more activated CD8+ T-cells and myeloid cells and amplifying inflammation, consistent with the “push/pull” model proposed by Shacklett et al[37]. Following initiation of ART and suppression of HIV RNA, CD8+CD107a expression and lytic activity may be restored if CD4+ T-cell levels are reconstituted to above 400 cells/ml in the periphery. If residual HIV replication or protein expression in the CNS elicits CXCL10, revitalized CD8+CD107a+ lytic T-cells would again migrate into CSF and CNS, destroying HIV expressing cells, reducing chemokine expression and halting progression of, if not reversing NCI. If however, virus persisted in the CNS and CD4+ T-cell levels were not reconstituted to >400 cells/μl following ART (and therefore unable to support CD8 lytic activity), trafficking of IFNγ expressing CD8+ T-cells to the CNS might increase, promoting inflammation without destruction of HIV infected cells and ultimately leading to indirect or bystander injury of neurons and HAND.

The major limitations to this study were the modest size and heterogeneity of the cohort with respect to time on ART and ART regimen at baseline, as well as inability to control for interval of HIV RNA suppression. Other potential covariats include HCV, substance abuse and level of ART adherence. However, the internal consistency of the data, despite diversity of individual T-cell response patterns and other limitations, speaks to the generalizibility of the findings.

Despite limitations, recognition that CD8+T-cells have the potential to either limit or promote neuroinflammation is significant with respect to understanding and management of HAND and may impact overall HV treatment strategy. Together, findings suggest that progressive NCI represents a convergence of innate and adaptive responses including CD8+T-cell IFNγ and IL-2 responses, activated monocytes, diminishing CD4 T-cell support for CD8 cytolytic activity, and high levels of beta chemokine expression in CSF. In addition to affirming the importance of complete viral suppression, the findings support early ART intervention for preservation of CD4 T-cells and thereby effective CD8+CTL immune surveillance in CNS, with prevention of neuropathogenesis.
